# Inhaled CD24-Enriched Exosomes (EXO-CD24) as a Novel Immune Modulator in Respiratory Disease

**DOI:** 10.3390/ijms25010077

**Published:** 2023-12-20

**Authors:** Shiran Shapira, Reut Schwartz, Sotirios Tsiodras, Amir Bar-Shai, Ariel Melloul, Sarah Borsekofsky, Michael Peer, Nimrod Adi, Ronan MacLoughlin, Nadir Arber

**Affiliations:** 1 Health Promotion Center and Integrated Cancer Prevention Center, Tel Aviv Sourasky Medical Center, Tel Aviv 6423906, Israel; shiransha@tlvmc.gov.il (S.S.); amirbs@tlvmc.gov.il (A.B.-S.); arielmelloul@gmail.com (A.M.); 2 Department of Molecular Genetics and Biochemistry, Sackler Faculty of Medicine, Tel Aviv University, Tel Aviv 6997801, Israel; 3 Sackler Faculty of Medicine, Tel Aviv University, Tel Aviv 6997801, Israel; reuts@tlvmc.gov.il (R.S.); nimroda@tlvmc.gov.il (N.A.); 4 Anesthesia and Intensive Care Unit, Tel Aviv Sourasky Medical Center, Tel Aviv 6423906, Israel; 5 4th Department of Internal Medicine, University General Hospital Attikon, Medical School, National and Kapodistrian University of Athens, 12462 Athens, Greece; sotirios.tsiodras@gmail.com; 6 Department of Pathology, Tel Aviv Sourasky Medical Center, Tel Aviv 6423906, Israel; sarahbo@tlvmc.gov.il; 7 Department of Chest Surgery, Tel Aviv Sourasky Medical Center, Tel Aviv 6423906, Israel; michaelpe@tlvmc.gov.il; 8 School of Pharmacy and Biomolecular Sciences, Royal College of Surgeons, D02 YN77 Dublin, Ireland; rmacloughlin@aerogen.com; 9 School of Pharmacy and Pharmaceutical Sciences, Trinity College, D02 PN40 Dublin, Ireland

**Keywords:** CD24, exosomes, small extracellular vesicles, exosomes, EXO-CD24, cytokine storm, ARDS, sepsis, asthma, pulmonary fibrosis

## Abstract

Acute Respiratory Distress Syndrome (ARDS) is a major health concern with urgent unmet need for treatment options. There are three million new ARDS cases annually, and the disease’s mortality rate is high (35–46%). Cluster of differentiation 24 (CD24), a long-known protein with multifaceted functions, is a small, heavily glycosylated, membrane-anchored protein which functions as an immune checkpoint control. CD24 allows for immune discrimination between Damage-Associated Molecular Patterns and Pathogen-Associated Molecular Patterns derived from pathogens. Exosomes are intraluminal vesicles which play an important role in intercellular communication. Exosomes offer the advantage of targeted delivery, which improves safety and efficacy. The safety and efficacy of EXO-CD24 is promising, as was shown in >180 ARDS patients in phase 1b/2a, phase 2b, and compassionate use. CD24 binds Damage-associated molecular patterns (DAMPs) and inhibits the activation of the NF-ĸB pathway, a pivotal mediator of inflammatory responses. In contrast to anti-inflammatory therapies that are cytokine-specific or steroids that shut down the entire immune system, EXO-CD24 acts upstream, reverting the immune system back to normal activity. Herein, the safety and efficacy of mEXO-CD24 is shown in murine models of several pulmonary diseases (sepsis, allergic asthma, Chronic Obstructive Pulmonary Disease(COPD), fibrosis). EXO CD24 can suppress the hyperinflammatory response in the lungs in several pulmonary diseases with a significant unmet need for treatment options.

## 1. Introduction

Acute Respiratory Distress Syndrome (ARDS) is a clinical syndrome of acute respiratory failure caused by diffuse lung inflammation and edema [1,2]. ARDS is a major health concern, with three million new cases being recorded annually and a high mortality rate ranging from 35% to 46% based on disease severity at the onset [3,4]. Rapid clinical deterioration can occur over a short period of 24–72 h [5,6]. This life-threatening disease is characterized by vascular leakage, leading to severe hypoxemia that often necessitates invasive mechanical ventilation. There is no medical therapy for ARDS [7]. Over the last three decades, a lung-protective strategy of ventilation remains the only disease-specific therapy shown to improve survival [7]. Several new and repurposed therapeutics were assessed in COVID-induced ARDS, with dexamethasone being the most promising [8]. 

Cytokine release syndrome (CSR) describes a variety of events that can ultimately lead to multi-organ failure and death [9]. CSR is an extreme inflammatory response characterized by a massive secretion of pro-inflammatory mediators that is disproportional and increases to a deleterious level in some patients [10,11]. As such, a therapeutic regimen that would dampen unwarranted immune processes while not interfering with anti-pathogenic immunity is of significant interest. 

Cluster Differentiation 24 (CD24) is a small, heavily glycosylated Glycosylphosphatidylinositol (GPI)-anchored protein which serves as an important immune check point inhibitor and immunomodulator [12]. CD24 plays a crucial role in facilitating immune discrimination between Damage-Associated Molecular Patterns (DAMPs), which are endogenous molecules that are constitutively expressed and released upon tissue damage or stress from damaged or dying cells, and Pathogen-Associated Molecular Patterns (PAMPs), molecular structures produced by microorganisms [13,14,15]. CD24 binds to DAMPs but not PAMPs, enabling the differentiation of “self” from “non-self” (e.g., bacteria, fungi, viruses). CD24, by binding to DAMPs, prevents their interaction with pattern recognition receptors (PRRs) (e.g., TLRs, NLRs), thus inhibiting the activation of the nuclear Factor-ĸB (NF-kB) pathway [14,15,16]. CD24 attenuates (NF-ĸB) activation, and therefore immune activation, by at least two mechanisms. CD24 binds to DAMPs, preventing them from binding to PRRs, thus inhibiting the activation of the NF-ĸB pathway, a key signaling pathway driving the production of cytokines and chemokines. Siglecs are distinct classes of PRRs that regulate immune cell functions. CD24, Siglec 10, and DAMPs form a trimolecular complex, resulting in the activation of the Siglec-10 signaling pathway. This pathway negatively regulates the activity of NF-ĸB through the phosphorylation of immunoreceptor tyrosine-based inhibition motif (ITIM) domains associated with SHP-1 and 2 [13,16,17]. This dual immune checkpoint inhibition provides tight regulation and potent anti-inflammatory activity [18]. In cases of bacterial pathogens, in addition to providing PAMPs and DAMPs, microbes may disrupt sialic acid-based pattern recognition to exacerbate innate immunity. Microbial sialidase exacerbates inflammation by removing sialic acid from CD24 so that it cannot be recognized by siglec-10 and therefore enhance the inflammatory response [19].

Exosomes are endogenous, endosomal, intraluminal, nano-vesicles (30–200 nm) with a reportedly high level of biocompatibility that play a vital role in intercellular communication [20]. Exosome-based delivery offers the advantage of targeted local delivery that should improve safety and efficacy. Their major benefits include tolerability and low immunogenicity due to their structural similarity to the cell membranes from which they originate [21]. Exosomes are the focus of significant ongoing clinical research involving evaluating their potential as diagnostic tools and carriers of therapeutic agents (cancer, cardiovascular, diabetic, graft-versus-host, neurological, orthopedic diseases) in 194 clinical trials. No drug based on exosomes has been regulatorily approved [22,23,24,25,26,27]. 

EXO-CD24 are exosomes genetically enriched with the CD24 glycoprotein [28]. EXO-CD24 was designed as a targeted therapy for hyperactive immune systems in the context of ARDS induced by COVID-19 [18,29].

The delivery of therapeutics directly to the lung via a nebulizer is well established in the critical care setting [30]. The aerosol-mediated delivery of exosomes has already been shown to facilitate therapeutic outcomes and potential advantages over intravenous administration [31,32]. Indeed, the aerosol-mediated delivery of exosomes has already been investigated as a means of improving distribution throughout the airways, ranging from the upper conducting airways down to the alveolar space [33]. Nebulizers are the most commonly used in the evaluation of the aerosol-mediated delivery of exosomes to date; however, no regulatory approved exosome–nebulizer combination has been approved yet [34].

In a phase Ib/IIa study (NCT04747574) involving 35 patients with COVID-19-induced ARDS, the safety of EXO-CD24 was demonstrated, as was its promising efficacy [18]. A phase IIb (NCT04902183) dose finding study in 91 patients in Greece confirmed this safety profile and again supported the potential for therapeutic efficacy. Further, in a separate study, again, efficacy and safety were also confirmed in 12 patients with severe ARDS [29]. An international, multi-center, randomized, quadri-blind clinical study of EXO-CD24 versus a placebo has recently opened for recruitment (NCT05947747).

Following on from this positive clinical work and the similarity in the underlying pathologies of many respiratory diseases, we hypothesized that EXO-CD24 may have applications in other disease models. Herein, we present selected preclinical data in various models of inflammatory and fibrotic lung disease. 

## 2. Results

### 2.1. Effect of EXO-mCD24 Compared to Dexamethasone in ARDS

We aimed to evaluate the effect of dexamethasone compared to that of EXO-mCD24 in the LPS-induced ARDS model. Dexamethasone was selected due to the utility it demonstrated in the RECOVERY trial [8]. 

**Figure 1:**
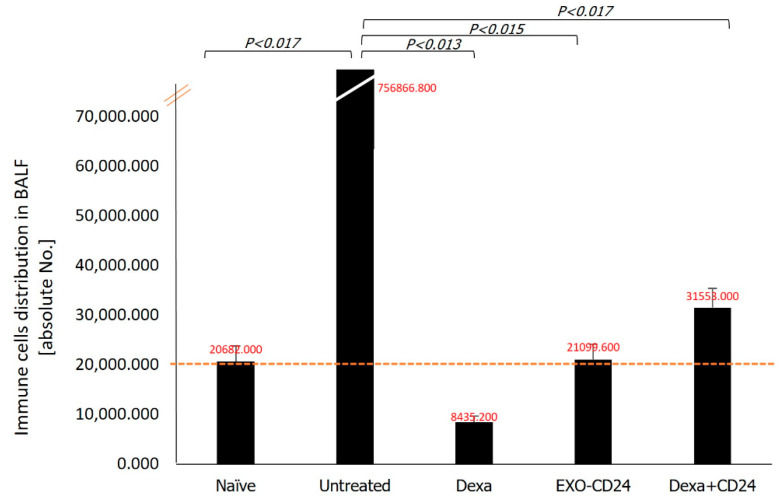
Cell composition in broncho alveolar lavage fluid. Total of Bronchial Alveolar Lavage Fluid (BALF) differential cell counting was performed by flow cytometry for T and B lymphocytes, eosinophils, neutrophils, dendritic cells, and monocytes/macrophages.

It was shown that EXO-mCD24 exosomes and dexamethasone ameliorated the clinical symptoms similarly. The total number of immune cells in the BALF was significantly increased (756,867 ± 141,150) following LPS stimulation, but it reverted back to normal levels (21,099 ± 3043) in the mEXO-CD24-treated mice (*p* < 0.015). In contrast, significantly reduced numbers (84,435 ± 1216), below normal levels, of immune cells were noted in the dexamethasone-treated mice, as can be seen in Figure 1.

**Figure 2:**
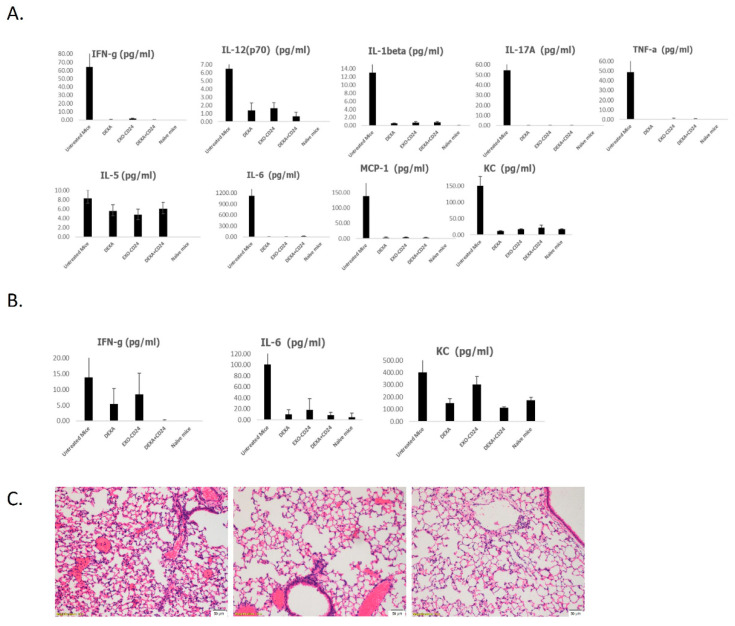
Inflammatory indices in a mice ARDS model. The levels of local (BALF, (**A**)) and systemic (serum, (**B**)) cytokines and chemokines were measured using the Luminex’s xMAP cytokine multiplex array. Data are represented as average ± SEM; n = 10 mice/group. (**C**) Histology. Left: moderate to severe lesion, including neutrophil infiltration, fibrin deposition, and alveolar wall thickening (H&E ×10). Middle: Dexa + EXO-CD24-treated animal, mild to moderate lesions, including decreased neutrophil infiltration, decreased fibrin deposition, and less alveolar wall thickening compared to the other treated groups (H&E. ×10). Right: naïve animal, very mild lesions (H&E. ×10).

Using Luminex immunoassays, the inflammatory response was noted to be similar for both drugs, as represented by the levels of the secreted cytokines and chemokines in the BALF (Figure 2A) and serum (Figure 2B).

Our histological analysis confirmed that all the LPS groups showed the infiltration of neutrophils into the BALF junction with a multifocal to coalescing pattern. Among the naïve animals, all but one showed very mild neutrophil infiltration, and their scores were markedly low (score 1.66). Our histopathology evaluation, at this short time point, demonstrated the potential of the combined treatment. The lung injury score was significantly lower (*p* = 0.013) in both the EXO-mCD24- and dexamethasone-treated groups individually when compared with all other groups (Figure 2C).

### 2.2. Effect of EXO-CD24 in Pseudomonas Aeruginosa-Induced Pulmonary Sepsis 

The aim of this study was to determine the immunomodulatory effects of EXO-mCD24 in inhibiting the inflammatory response and the clinical symptoms of bacteria-induced ARDS. Specifically, we aimed to evaluate the potential efficacy of the aerosolized exosomes in the downregulation of the “cytokine storm” and consequent abrogation of lung injury. The recognition of bacterial endotoxins through pattern recognition receptors induces an initial systemic pro-inflammatory phase characterized by a massive release of cytokines, acute-phase proteins, and reactive oxygen species. In spite of appropriate effective antibiotics, *Pseudomonas aeruginosa* is associated with very high rates of attributable mortality in acute pulmonary infections [35,36].

The evaluation of histopathological changes and grade of inflammation in the lungs included a comparison between the treated, control, and naïve animals. A semi-quantitative analysis showed that the scores of all the EXO-mCD24 treated groups were lower and statistically significant (*p* ≤ 0.005) compared to the scores of the parallel saline groups.

Our digital analysis of cellularity (number of cells) indicated a similar trend as the semi-quantitative grading. The mean cells’ numbers of all the EXO-mCD24-treated groups were lower than those of the parallel saline groups. The difference between all groups was statistically significant (*p* ≤ 0.05). 

An evaluation of pathological changes and a quantitative analysis of cellularity were carried out on 41 H&E-stained lung samples. A moderate to severe inflammatory reaction was observed, characterized by cellular inflammation (predominantly neutrophils) and edema. The semi-quantitative analysis and the digital morphometry of the inflammatory grade revealed a similar pattern of lower significant scores in the EXO-mCD24 groups compared to the parallel saline groups.

**Figure 3:**
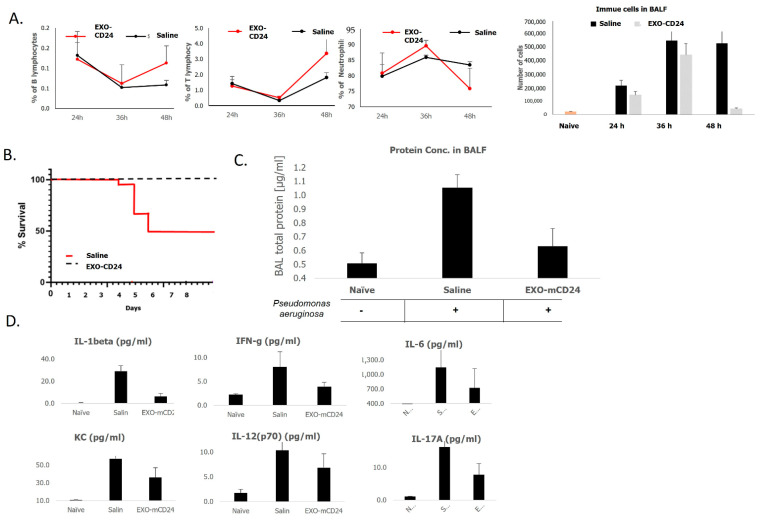
(**A**). Cell count in mice’ broncho alveolar lavage fluid. Cells from the BALF tissues were analyzed for the differential cell counts of BALF cells by flow cytometry for eosinophils, B cells, T cells, and neutrophils. (**B**). Survival of mice. Pulmonary sepsis was induced, followed by an inhalation treatment with vehicle or 10^10^ EXO-mCD24/mouse. (**C**). BALF total protein concentration. The total protein concentration of cell-free BALF fluid was determined. The graph depicts the mean protein concentration values, and standard deviations are indicated by error bars. (**D**). The levels of secreted cytokines and chemokines were measured using the Luminex’s xMAP cytokine “Multi-plex” array. Data are represented as average ± SEM; n = 10 mice/group.

The cell distribution in the BALF tissues revealed that EXO-mCD24 increased lymphocyte count compared to the untreated animals (B lymph 0.113 ± 0.04% vs. 0.058 ± 0.01%; T lymph 3.4 ± 1.8% vs. 1.8 ± 0.3%) and reduced the percentage of neutrophils (75.8 ± 6.5 vs. 83.4 ± 1.0%) (Figure 3A). 

Regarding survival, the mice were followed-up for survival up to one week. The Cox regression showed that while 50% of mice from the control group died after 1 week, no deaths were noted in that time frame among the mice that were treated with EXO-mCD24 (*p* < 0.05). 

The total concentration of proteins produced by the *P. aeruginosa* challenge increased by nearly 210% in the BALF. It was significantly reduced after 36 h in the EXO-mCD24-treated animals (*p* < 0.05), almost reaching base level, similar to the levels in the naïve mice (NS).

Cytokine and chemokine analyses in the BALF showed a significant reduction in levels 48 h after *P. aeruginosa* challenge in the EXO-mCD24-treated group after only four treatments. 

#### 2.2.1. Effect of EXO-CD24 in an Ovalbumin (OVA)-Induced Type I Inflammation Model of Asthma

Basically, the allergic airway inflammation was induced by intraperitoneal sensitization and intratracheal challenge with ovalbumin. The model provided here mimics acute asthma characteristics, including excessive mucus production, airway hyperresponsiveness, and airway inflammation. Differential cell counts, % of BALF cells, was analyzed by flow cytometry, and the results showed a significant change in the percentage of B and T lymphocytes in the EXO-mCD24-treated animals compared to the untreated animals (Table 1).

**Figure 4:**
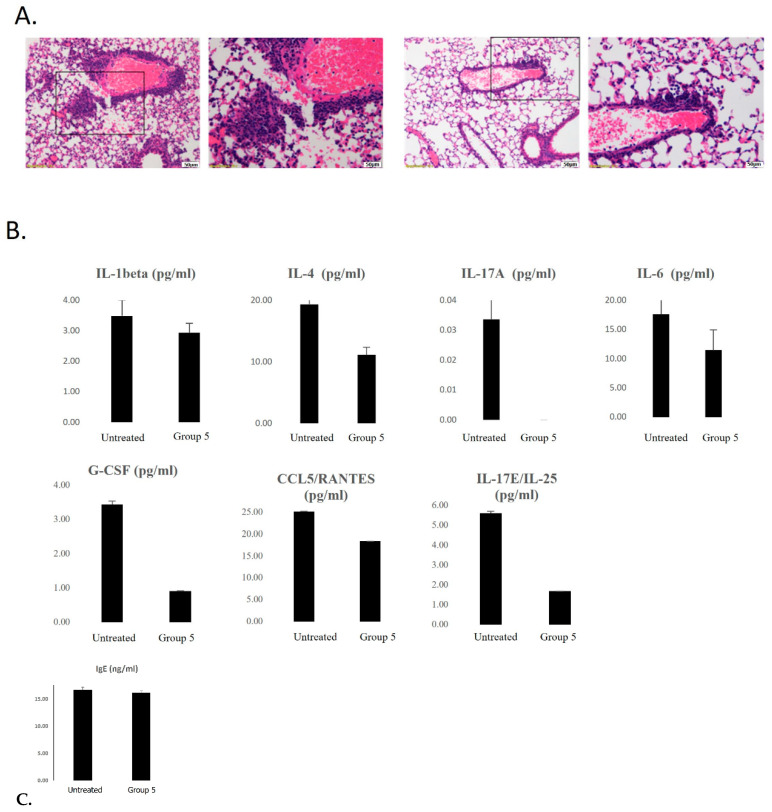
Histological images comparing inflammatory infiltrates between the untreated and EXO-mCD24-treated mice with induced Asthma. (**A**) Left: untreated mouse. Moderate inflammatory infiltration. Rectangle—the area enlarged in the following figure (H&E ×10). Right: treated mouse. Mild inflammatory infiltration. Rectangle—the area enlarged in the following figure (H&E ×10). (**B**) The levels of secreted cytokines and chemokines were measured using the Luminex’s xMAP cytokine multiplex array. (**C**) The levels of IgE were measured. Data are represented as average ± SEM; n = 10 mice/group.

Overall, all the emphysema-induced animals displayed the same type of inflammation, characterized by a peri-bronchiolar (middle-sized) and a peri-vascular cellular infiltration composed of a mixed population of macrophages, lymphocytes, eosinophils, and mast cells. Necrosis was found in the respiratory epithelium of the affected bronchioles and in endothelial cell blood vessels. Edema was found along middle and large sized vessels and emphysema was noted in the peripheral parenchyma (ruptured alveoli). This type of pathology was identified to be a type 1 hypersensitivity reaction that is consistent with the allergic reaction in asthma (Figure 4A). 

While the inflammatory cell population was consistent, inflammation was significantly lower in group 5 (mice that were treated with inhalations before and during challenges (Table 2) compared to the other groups. Scores were high in groups 2–4 (~2.7) and significantly lower (*p* ≤ 0.01) in group 5 (1.9).

The edema scores were relatively similar, but the lowest was noted to be in group 5 (0.9). The emphysema and necrosis scores were similar in all experimental groups. Reduced inflammatory cytokine and chemokine levels in the BALF from the EXO-mCD24-treated animals compared to the untreated animals were observed for some cytokines/chemokines (Figure 4B). However, no differences were observed between the groups for IgE (Figure 4C). These findings suggest that EXO-mCD24 may attenuate the severity of the non-eosinophilic form of asthma, an asthma phenotype which has no effective treatment nowadays. 

#### 2.2.2. Bleomycin-Induced Pulmonary Fibrosis (PF)

**Figure 5:**
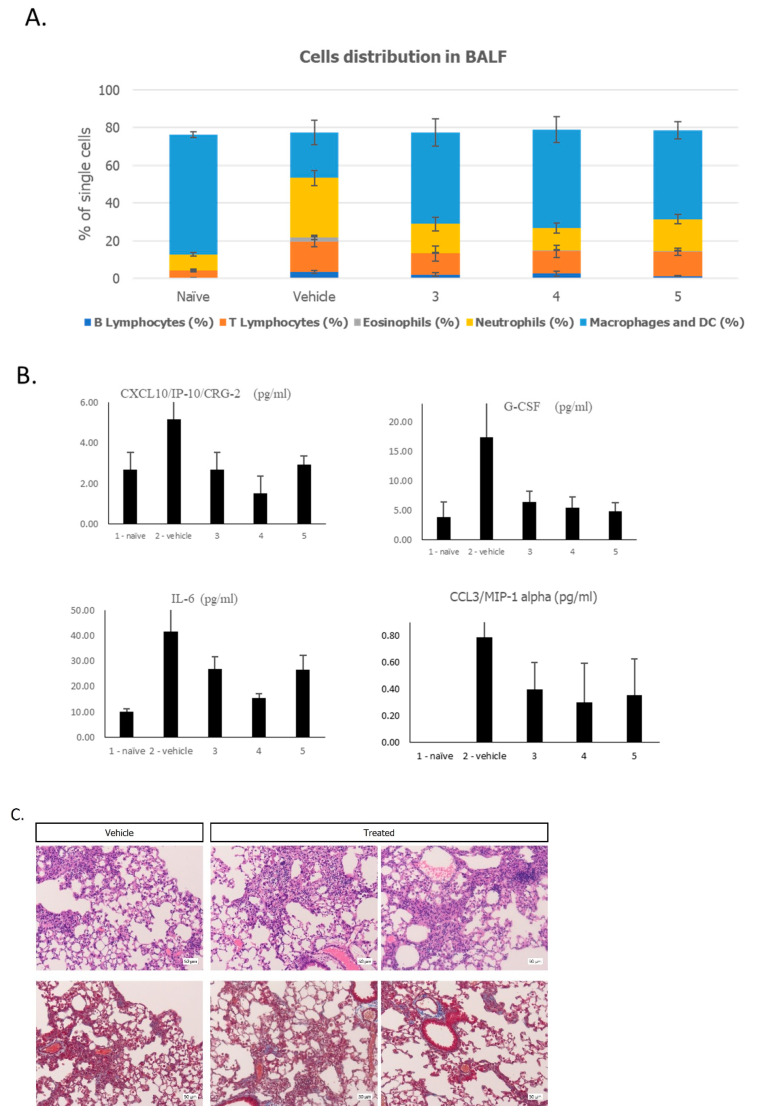
(**A**) Cell composition in broncho alveolar lavage fluid. Bronchial Alveolar Lavage Fluid (BALF) differential cell counting was performed by flow cytometry for T and B lymphocytes, eosinophils, neutrophils, dendritic cells, and monocytes/macrophages. (**B**) The levels of secreted cytokines and chemokines were measured using the Luminex’s xMAP cytokine “Multi-plex” array. Data are represented as average ± SEM; n = 10 mice/group. (**C**) Representative pictures of the affected areas in the lungs. ×20; H&E, MT (Masson’s trichrome). Upper panel: histopathological findings (H&E); bottom panel: Masson’s trichrome staining for fibrosis.

Bleomycin-induced PF is the best characterized and most widely used pulmonary fibrosis model. The results of our flow cytometry analysis (Figure 5A) for cell distribution in the BALF show that the three EXO-mCD24 treatment regimens were similar, and the trend of change compared to the PF-induced control group (group 2) was clear, with the percentage of macrophages and dendritic cells increasing, and the percentage of B lymphocytes, T lymphocytes, eosinophils, and neutrophils decreasing.

In addition, a reduction in secreted pro-inflammatory cytokines and chemokines such as CXCL10/IP-10/CRG-2, G-CSF, CCL3/MIP-1α, and IL-6 was shown in all the EXO-mCD24 treatment groups compared to the vehicle controls (Figure 5B). In contrast to the results presented above, at this timepoint, there was no improvement in lung histopathological findings and fibrosis among the treated groups (Figure 5C).

## 3. Discussion

The current study evaluated the utility of EXO-mCD24 across a variety of high-burden respiratory diseases. In this study, it was demonstrated that EXO-CD24 increased survival in an animal model of pulmonary sepsis and that it had positive effects in a model of ovalbumin type I-induced allergic asthma, as well as a model of bleomycin-induced pulmonary fibrosis. In addition to the evidence gathered in more than 180 ARDS patients treated with EXO-CD24, these standardized pre-clinical studies further highlight its potential [18,29]. In the LPS-induced ARDS mouse model, EXO-mCD24 was superior to dexamethasone, primarily by enabling the rapid reversion of the immune system back to normal activity, which allows for a more robust response to pathogen clearance.

The reason behind the superiority of CD24 over steroids lies in its ability to selectively modulate the host response to DAMPs without interfering with PAMPs’ immune recognition, which is crucial for an effective immune response. EXO-CD24, unlike steroids, does not shut down the entire immune system. CD24 helps in regulating immune response, preventing excessive activation while still allowing for the effective clearance of pathogens. EXO-CD24 achieves this by tightly regulating the NF ĸB pathway and reverting it back to normal activity without interfering with pathogen clearance. It therefore enables the rapid reversion of the immune system back to normal activity, which may assist with normal innate immune response activities such as pathogen clearance.

EXO-mCD24 increased survival in an animal model of pulmonary sepsis. EXO-CD24 has positive effects in a model of ovalbumin type I inflammation for allergy-induced asthma, as well as in bleomycin-induced pulmonary fibrosis. In the LPS-induced ARDS mouse model, EXO-mCD24 was superior to dexamethasone, mainly by enabling the rapid reversion of the immune system back to normal activity, which allows for a more robust response to pathogen clearance.

The cytokine storm is the main complication that leads to a wide range of respiratory and systemic diseases. In light of its effectiveness in a diverse range of preclinical models, as shown here, EXO-CD24 may serve as a platform with therapeutic potential in a variety of diseases with urgent unmet treatment need. Following on from the promising clinical results in mild, moderate, and severe patients, ARDS is the first indication for a placebo-controlled trail [18,29].

Our results are consistent with those reported for CD24Fc, a recombinant fusion protein composed of the extracellular domain of CD24, linked to a human immunoglobulin G1 (IgG1) Fc domain. CD24Fc reduced lung pathology and the incidence of viral-induced pneumonia in an animal model [37].

EXO-CD24 is hypothesized to be a promising treatment for other hyperinflammatory pulmonary diseases. The recent in vivo studies regarding different applications (pulmonary sepsis, ARDS, allergic asthma, pulmonary fibrosis) strengthen the clinical data of EXO-CD24 in ARDS, its superiority over steroids, and the use of EXO-CD24 as a platform for urgent unmet treatment needs.

Clinical efficacy must still be confirmed in the ongoing clinical studies involving the use of EXO-CD24 versus a placebo.

## 4. Materials and Methods

### 4.1. EXO-mCD24 Manufacture

EXO-mCD24 is a homologous mouse-derived product that was generated to examine the efficacy of the drug in mouse inflammatory disease models. It is based on exosomes enriched with mCD24 derived from Expi293F™ expression system (Gibco^TM^, Rehnium, Modiin, Israel, A14527), a high-yield transient expression system based on suspension-adapted Human Embryonic Kidney (HEK) cells. Briefly, cells were subcultured and expanded until the cells reached a density of approximately 3–5 × 10^6^ viable cells/mL. On the day prior to transfection (Day −1), Expi293F™ culture cells were seeded to a final density of 2.5–3 × 10^6^ viable cells/mL and were allowed to grow overnight. On the next day (Day 0), cell viability and density were determined, and the cells were diluted to a final concentration of 75 × 10^6^ cells/30 mL culture. ExpiFectamine™ 293/plasmid DNA complexes were prepared (Total plasmid DNA of 1.0 µg per mL of culture volume Total plasmid DNA of 1.0 µg per mL of culture volume) and added to the cells. At 18–22 h post transfection, ExpiFectamine™ (Gibco^TM^, Rehnium, Modiin, Israel, A14524) 293 Transfection Enhancer 1, and ExpiFectamine™ 293 Transfection Enhancer 2 were added to the flasks, and the cells were incubated in a 37 °C incubator with a humidified atmosphere of 8% CO_2_ with shaking (125 rpm). After 5 days, the cells were harvested, and exosomes were purified. 

### 4.2. EXO-mCD24 Purification

Biofluid was collected and centrifuged at 3000× *g* for 15 min (4 °C) to remove cells and cell debris. The supernatant was then filtered using a PES filter with a 0.22 µm pore size. Exosomes were purified using a PEG/NaCl solution. A volume of 5 mL PEG/NaCl solution (30% PEG, 1.5 M NaCl) was added per 10 mL biofluid. Tubes were mixed by gentle inversion and refrigerated overnight (12–16 h; at least 12 h). The next day, the PEG/biofluid mixture was centrifuged at 4000 rpm for 60 min, at 4 °C, and the supernatant was aspirated. The residual PEG solution was removed by centrifugation at 4000 rpm for 5 min, and the aspiration of all traces of fluid was performed. The pellets from all tubes were resuspended in saline (0.9% sodium chloride), combined, homogenized using a 50 mL pipette, and transferred to a dialysis tube. Dialysis was performed against freshly prepared PBS at a volume ratio of at least 1:270 for at least 16 h at 4 °C using a membrane cut-off of 100 kDa. The dialysis membrane was composed of cellulose ester membrane, biotech-grade (product: Spectra Por G235037 Float-A-Lyzer G2 Dialysis Device or 88253 SPECTRA/POR^®^—BIOTECH CE, 16 mm width tubing, 1,000,000 MWCO, Fisher Scientific, Waltham, MA, USA). The exosome suspension was then transferred to Amicon^®^ centrifugal filter tubes (100 kDa MW) and centrifuged at 15 °C, at 4000–4500 rpm, until the required concentration was reached.

### 4.3. Animal Models of Lung Disease

#### 4.3.1. Lipopolysaccharide (LPS)-Induced ARDS

Female, 8-week-old C57/Bl mice were purchased from Envigo (Jerusalem, Israel), anesthetized (100 mg/kg of ketamine/xylazine mixture) and orally intubated using a sterile plastic catheter, and challenged with an intratracheal instillation of 800 µg LPS (*E. coli* 055:B5, Chemcruz, Heidelberg, Germany), dissolved in 50 µL PBS, to induce ARDS. 

A total of 5 groups of mice were randomized (Table 3) and daily doses of EXO-mCD24 (10^10^) were given to them in inhalation exposure chambers. Dexamethasone 100 µg/mouse was administered via IV injection 10 min before the inhalation. Treatment was initiated three hours after LPS administration. The study was terminated 72 h after LPS challenge and serum, and BALFs and tissues were collected for analysis. The Mann–Whitney U statistical test was used to compare between animal groups.

#### 4.3.2. *Pseudomonas aeruginosa*-Induced Pulmonary Sepsis

Female, 8-week-old C57bl mice (n = 54) were purchased from Envigo, anesthetized and orally intubated using a sterile plastic catheter, and challenged with an intratracheal instillation of *Pseudomonas aeruginosa* bacteria [2 × 10^6^ colony forming units (CFU) of PAO1 strain, ATCC BAA-47 in 50 µL saline]. Briefly, the animals were laid down at an angle of 45 degrees, and with the help of fine tweezers, their mouths were opened, and the oral cavity was illuminated with an otoscope at ×5 magnification. In this way, the trachea was identified, and a 24 G catheter was inserted until it reached the carina. The catheter was pulled posteriorly 5 mm, and the bacterial fluid was sprayed into the lungs. Dosing (1 × 10^10^ mCD24-exosomes/mouse) was performed in an inhalation cage for 20 min, starting 2 h after the induction of the disease and then twice a day thereafter (Table 4). The animals were weighed daily. Six mice from each group were sacrificed for sample collection, after 24, 36, and 48 h. BALF and lung tissues were used to evaluate inflammation; differential cell count was evaluated by FACS, and the multiplex assay was used for the evaluation of cytokines and chemokines. BALF total protein concentration (Bradford) was also measured, and a histopathological analysis was conducted. The remaining animals, from groups 4 and 7 (n = 6), were followed-up for survival up to one week. Survival across treatment groups was monitored. Six mice survived for up to one week. The Mann–Whitney U statistical test was used to compare between the animal groups. 

#### 4.3.3. Ovalbumin (OVA)-Induced Type I Inflammation Model of Asthma

BALB/c female mice were sensitized with 100 µg OVA in 2 mg aluminum hydroxide by intraperitoneal injections (200 µL) performed once a week (on days 0, 7 and 14), followed by OVA challenge (exposure for 20 min to nebulized 4% OVA) for four consecutive days (days 25, 26, 27 and 28). EXO-mCD24 was administered in an inhalation cage for 20 min on days 22, 23, and 24, and/or during the challenges on days 26, 27, and 28. Blood was collected 24 h after challenge (day 29). Mice were sacrificed, and BALF and lung tissue were obtained. Differential cell counts (% of BALF cells) was analyzed by flow cytometry; the multiplex assay was used for the evaluation of cytokines, chemokines, and IgE (ThermoFisher, Rhenium, Modiin, Israel).

The Mann–Whitney U statistical test was used to compare between the animal groups. 

#### 4.3.4. Bleomycin-Induced Pulmonary Fibrosis

Induction was achieved with a single intratracheal injection of 3 U/kg bleomycin sulfate solution in C57bl female mice. Pulmonary fibrosis developed over two weeks. 

EXO-CD24 treatment (10^10^ exosomes/200 µL) was started 1 or 5 days after induction and continued for 5–10 days (Table 5). The treatment was administered by inhalation in a dedicated exposure chamber connected to a nebulizer for 20 min. On day 14, blood samples were collected, mice were sacrificed, and BALF and lung tissues were obtained for inflammatory markers.

### 4.4. Bronchoalveolar Lavage (BAL)

BAL is a technique used to collect the contents of the airways. The animals were laid on their backs, their necks were opened, and the trachea was exposed. A 24 G catheter was inserted into the trachea, and 1 mL of saline was instilled into the lung, lavaged, and removed again. The cells in the lung fluid were separated and examined via flow cytometry to count and quantify the total number of cells and their types. Cells positive for CCR3 and negative for CD3/CD45R were typed as eosinophils. Cells negative for CCR3 and negative for CD3/CD45R were typed as neutrophils. Cells positive for CD3/CD45R and negative for MHC-II were typed as T cells. Cells positive for CD3/CD45R and positive for MHC-II were typed as B cells. Large cells positive for CD11c were typed as macrophages and DCs. FACS analysis was performed using the CytoFLEX instrument (Beckman Coulter, Brea, CA, USA).

### 4.5. Histopathological Score

#### 4.5.1. LPS-Induced ARDS and Bleomycin-Induced Pulmonary Sepsis

The lungs of the mice were harvested, fixed in 4% formaldehyde, trimmed, placed in cassettes, and processed routinely for paraffin embedding. Four-micron-thick paraffin sections were cut, put on glass slides, and stained with hematoxylin and eosin (H&E). All steps were performed and reported based on OECD-GLP principles. A semi-quantitative analysis for Acute Lung Injury (ALI) was performed using a modified severity scoring scale of 0–2 (Table 6) [17].

Only septal thickening that is equal to or greater than twice the normal diameter was considered. The analysis was based on measurements in at least 20 injured lung areas using objective ×40 magnification. The segmentation of the cells’ nuclei was performed by morphological, color-based, and brightness-based segmentation using MATLAB software, 9.7 R2019b. The results were subjected to statistical analysis using the Kruskal–Wallis test to evaluate the differences among the non-naïve groups.

#### 4.5.2. Ovalbumin (OVA)-Induced Asthma

Histological evaluation was performed and reported based on the OECD-GLP Principles. The H&E-stained slides were examined, described, and scored by the study pathologist using a semi-quantitative, five-point grading system (0–4) for the severity of the pathological changes. The criteria for each grade are listed in Table 7.

The histopathological evaluation included a comparison between the treated and control or naïve animals. Pathological findings were described, scored, and demonstrated in representative histological pictures. The results of the treated and untreated groups were subjected to the Kruskal–Wallis test. *p* > 0.05 was considered significant.

### 4.6. Cytokine/Chemokine Analysis

MILLIPLEX^®^ Mouse Cytokine/Chemokine Magnetic Bead Panels (Millipore, Merck, Rosh-Ha’ayin, Israel) based on the Luminex^®^ xMAP^®^ technology (Merck, Rosh-Ha’ayin, Israel) were used. Briefly, this involved using internally color-coded microspheres with multiple fluorescent dyes. Through using precise concentrations of these dyes, distinctly colored bead sets of 80–6.45 μm magnetic polystyrene microspheres were created, each of which was coated with a specific capture antibody. After an analyte from the BALF or plasma test samples was captured by the bead, a biotinylated detection antibody was introduced. The reaction mixture was then incubated with a Streptavidin–PE conjugate, the reporter molecule, to complete the reaction on the surface of each microsphere. Luminex^®^ instruments were used to acquire and analyze data. Each individual microsphere was identified, and the result of its bioassay was quantified based on fluorescent reporter signals. Median Fluorescence Intensity (MFI) data, using a 5-parameter logistic method for calculating the cytokine/chemokine concentrations in the samples, was analyzed. The tested analytes included IL-13; IL-17A; IL-12 (p70); IL-10; IL-5; IL-6; IL-1 beta; TNF-alpha; IFN-gamma; JE/MCP-1/CCL2; and KC/GRO-a/CXCL1.

### 4.7. Ethical Committee Approval

All studies were performed under the approval of “The Israeli Board for Animal Experiments” and were in compliance with “The Israeli Animal Welfare Act” and Ethics Committee. The Ethics Committee Approval Numbers for the studies described above are as follows: IL-20-12-591, IL-2202-110-5, IL-21-1-2, and IL-20-6-203.

### 4.8. Statistical Analysis

In the pre-clinical animal studies, the Mann–Whitney U statistical test was used to compare between the animal groups.

For other tests, statistical analysis was conducted using the Kruskal–Wallis test for the semi quantitative data, and an ANOVA was conducted for the digital analysis data. The significance level was *p* ≤ 0.05 in both tests.

## Figures and Tables

**Table 1:** Differential cell counts (% of BALF cells).

Group	Emphysema Induction	Treatment	B Lymphocytes (%)	T Lymphocytes (%)
1	n/a	n/a	0.249 ± 0.043	2.214 ± 0.455
2	OVA	n/a	0.537 ± 0.109	4.684 ± 0.504
3	OVA	inhalations before challenges (days 22, 23, 24) (total of three inhalations)	0.27 ± 0.042 (*p* < 0.04)	3.233 ± 0.266 (*p* < 0.01)
4	OVA	inhalations during challenges (day 26, 27, 28) (total of three inhalations)	0.57 ± 0.11	3.9 ± 0.53
5	OVA	inhalations before challenges (days 22, 23, 24) and during challenges (days 26, 27, 28) (total of six inhalations)	0.255 ± 0.031 (*p* < 0.02)	3.043 ± 0.267 (*p* < 0.01)

n/a—Not applicable. OVA—ovalbumin.

**Table 2:** Study design for EXO-mCD24 in OVA-induced murine asthma.

Group No.	OVA Sensitization	EXO-mCD24/Vehicle	OVA Sensitization
1	None	None	None
2	yes	Vehicle	inhalations before challenges (days 22, 23, 24) and during challenges (days 26, 27, 28) (total of six inhalations)
3	yes	10^10^	inhalations before challenges (days 22, 23, 24) (total of three inhalations)
4	yes	10^10^	inhalations during challenges (day 26, 27, 28) (total of three inhalations)
5	yes	10^10^	inhalations before challenges (days 22, 23, 24) and during challenges (days 26, 27, 28) (total of six inhalations)

**Table 3:** EXO-mCD24 versus dexamethasone.

Group No.	C57bl Mice No/Gender	ARDS Model Induction	Treatment
1	10/F	LPS	saline
2	10/F	LPS	dexamethasone
3	10/F	LPS	EXO-mCD24
4	10/F	LPS	dexamethasone + EXO-mCD24
5	3/F	none	none

**Table 4:** Study design for EXO-mCD24 in a murine model of pulmonary sepsis.

Group No.	N	Induction of Pulmonary Sepsis	Treatment/Treatment Time
1	6	None	None
2	6	PAO1	Saline/2 and 12 h
3	6	PAO1	Saline/2, 12 and 24 h
4	12	PAO1	Saline/2, 12, 24 and 36 h
5	6	PAO1	10^10^ EXO-mCD24/2 and 12 h
6	6	PAO1	10^10^ EXO-mCD24/2,12 and 24 h
7	12	PAO1	10^10^ EXO-mCD24/2, 12, 24 and 36 h

**Table 5:** Study design for EXO-mCD24 in bleomycin-induced pulmonary fibrosis (PF).

Group No.	Bleomycin PF Induction	Treatment	Treatment Schedule
1	-	none	none
2	+	Saline	10 days of inhalation beginning 24 h after PF induction
3	+	EXO-mCD24 (10^10^)	5 days of inhalation beginning 24 h after PF induction
4	+	EXO-mCD24 (10^10^)	5 days of inhalation beginning 5 days after PF induction
5	+	EXO-mCD24 (10^10^)	10 days of inhalation beginning 24 h after PF induction

**Table 6:** Grade of inflammation (American Thoracic Society 2011).

Feature	Score 0	Score 1	Score 2
Neutrophils	Not visible within the field	1–5 neutrophils	>5 neutrophils
Fibrin	Not visible within the field	A single well-formed band within the air spaces	Fibrin
Thickened alveolar walls	<2	Thickened alveolar walls	<2

**Table 7:** Semi-quantitative histological scoring.

Feature	Grade 0	Grade 1	Grade 2	Grade 3	Grade 4
Cellular inflammation	The tissue appears normal, without any changes at all	Minimal	Mild	Moderate	Severe
Inflammatory cell differentiation in %	Lymphocytes, macrophages, neutrophils, eosinophils, mast cells				
Necrosis	The tissue appears normal, without any changes at all	Minimal	Mild	Moderate	Severe
Edema (intra-alveolar)	The tissue appears normal, without any edema	Minimal	Mild	Moderate	Severe
Emphysema	The tissue appears normal, without any changes at all	Minimal	Mild	Moderate	Severe

## Data Availability

Data is contained within the article.

## References

[1] Gorman E.A., O’Kane C.M., McAuley D.F. (2022). Acute respiratory distress syndrome in adults: Diagnosis, outcomes, long-term sequelae, and management. Lancet.

[2] Ramji H.F., Hafiz M., Altaq H.H., Hussain S.T., Chaudry F. (2023). Acute Respiratory Distress Syndrome; A Review of Recent Updates and a Glance into the Future. Diagnostics.

[3] Fan E., Brodie D., Slutsky A.S. (2018). Acute Respiratory Distress Syndrome: Advances in Diagnosis and Treatment. JAMA.

[4] Parcha V., Kalra R., Bhatt S.P., Berra L., Arora G., Arora P. (2021). Trends and Geographic Variation in Acute Respiratory Failure and ARDS Mortality in the United States. Chest.

[5] Sheard S., Rao P., Devaraj A. (2012). Imaging of acute respiratory distress syndrome. Respir. Care.

[6] Jameson J.L., Kasper D.L., Hauser S.L., Longo D.L., Loscalzo J. (2018). Harrison’s Principles of Internal Medicine.

[7] Meyer N.J., Gattinoni L., Calfee C.S. (2021). Acute respiratory distress syndrome. Lancet.

[8] RECOVERY Collaborative Group (2021). Dexamethasone in Hospitalized Patients with COVID-19. N. Engl. J. Med..

[9] Ragab D., Eldin H.S., Taeimah M., Khattab R., Salem R. (2020). The COVID-19 Cytokine Storm; What We Know So Far. Front. Immunol..

[10] Fajgenbaum D.C., June C.H. (2020). Cytokine Storm. N. Engl. J. Med..

[11] Sinha P., Matthay M.A., Calfee C.S. (2020). Is a “Cytokine Storm” Relevant to COVID-19?. JAMA Intern. Med..

[12] Liu Y., Zheng P. (2007). CD24: A genetic checkpoint in T cell homeostasis and autoimmune diseases. Trends Immunol..

[13] Liu Y., Chen G.-Y., Zheng P. (2009). CD24-Siglec G/10 discriminates danger-from pathogen-associated molecular patterns. Trends Immunol..

[14] Liu Y., Zheng P. (2023). CD24-Siglec interactions in inflammatory diseases. Front. Immunol..

[15] Wang X., Liu M., Zhang J., Brown N.K., Zhang P., Zhang Y., Liu H., Du X., Wu W., Devenport M. (2022). CD24-Siglec axis is an innate immune checkpoint against metaflammation and metabolic disorder. Cell Metab..

[16] Chen G.-Y., Tang J., Zheng P., Liu Y. (2009). CD24 and Siglec-10 selectively repress tissue damage-induced immune responses. Science.

[17] Sabat R., Grütz G., Warszawska K., Kirsch S., Witte E., Wolk K., Geginat J. (2010). Biology of interleukin-10. Cytokine Growth Factor Rev..

[18] Shapira S., Ben Shimon M., Hay-Levi M., Shenberg G., Choshen G., Bannon L., Tepper M., Kazanov D., Seni J., Lev-Ari S. (2022). A novel platform for attenuating immune hyperactivity using EXO-CD24 in COVID-19 and beyond. EMBO Mol. Med..

[19] Chen G.Y., Chen X., King S., Cavassani K.A., Cheng J., Zheng X., Liu Y. (2011). Preserving Sialic Acid-dependent Pattern Recognition by CD24-Siglec G. Interaction for Therapy of Polybacterial Sepsis. Nat. Biotechnol..

[20] Jella K.K., Nasti T.H., Li Z., Malla S.R., Buchwald Z.S., Khan M.K. (2018). Exosomes, Their Biogenesis and Role in Inter-Cellular Communication, Tumor Microenvironment and Cancer Immunotherapy. Vaccines.

[21] Elliott R.O., He M. (2021). Unlocking the Power of Exosomes for Crossing Biological Barriers in Drug Delivery. Pharmaceutics.

[22] Colombo M., Raposo G., Théry C. (2014). Biogenesis, secretion, and intercellular interactions of exosomes and other extracellular vesicles. Annu. Rev. Cell Dev. Biol..

[23] Huda M.N., Nafiujjaman M., Deaguero I.G., Okonkwo J., Hill M.L., Kim T., Nurunnabi M. (2021). Potential Use of Exosomes as Diagnostic Biomarkers and in Targeted Drug Delivery: Progress in Clinical and Preclinical Applications. ACS Biomater. Sci. Eng..

[24] Chen L.W., Zhu L., Xu Z., Liu Y., Li Z., Zhou J., Luo F. (2022). Exosomes as Drug Carriers in Anti-Cancer Therapy. Front. Cell Dev. Biol..

[25] Hanjani N.A., Esmaelizad N., Zanganeh S., Gharavi A.T., Heidarizadeh P., Radfar M., Omidi F., MacLoughlin R., Doroudian M. (2022). Emerging role of exosomes as biomarkers in cancer treatment and diagnosis. Crit. Rev. Oncol. Hematol..

[26] Vahabi A., Rezaie J., Hassanpour M., Panahi Y., Nemati M., Rasmi Y., Nemati M. (2022). Tumor Cells-derived exosomal CircRNAs: Novel cancer drivers, molecular mechanisms, and clinical opportunities. Biochem. Pharmacol..

[27] Kalluri R., LeBleu V.S. (2020). The biology, function, and biomedical applications of exosomes. Science.

[28] Tsioulos G., Grigoropoulos I., Moschopoulos C.D., Shapira S., Poulakou G., Antoniadou A., Boumpas D., Arber N., Tsiodras S. (2022). Insights into CD24 and Exosome Physiology and Potential Role in View of Recent Advances in COVID-19 Therapeutics: A Narrative Review. Life.

[29] Green O., Shenberg G., Baruch R., Argaman L., Levin T., Michelson I., Hadary R., Isakovich B., Golos M., Schwartz R. (2023). Inhaled Exosomes Genetically Manipulated to Overexpress CD24 (EXO-CD24) as a Compassionate Use in Severe ARDS Patients. Biomedicines.

[30] MacLoughlin R., Martin-Loeches I. (2023). Not all nebulizers are created equal: Considerations in choosing a nebulizer for aerosol delivery during mechanical ventilation. Expert. Rev. Respir. Med..

[31] Brave H., MacLoughlin R. (2020). State of the Art Review of Cell Therapy in the Treatment of Lung Disease, and the Potential for Aerosol Delivery. Int. J. Mol. Sci..

[32] Frohlich E. (2021). Therapeutic Potential of Mesenchymal Stem Cells and Their Products in Lung Diseases-Intravenous Administration versus Inhalation. Pharmaceutics.

[33] Gonzalez H., McCarthy S., Masterson C., Byrnes D., Sallent I., Horan E., Elliman S.J., Vella G., Prina-Mello A., Silva J.D. (2023). Nebulised mesenchymal stem cell derived extracellular vesicles ameliorate *E. coli* induced pneumonia in a rodent model. Stem Cell Res. Ther..

[34] Woods N., MacLoughlin R. (2020). Defining a Regulatory Strategy for ATMP/Aerosol Delivery Device Combinations in the Treatment of Respiratory Disease. Pharmaceutics.

[35] Pachori P., Gothalwal R., Gandhi P. (2019). Emergence of antibiotic resistance Pseudomonas aeruginosa in intensive care unit: A critical review. Genes Dis..

[36] Sathe N., Beech P., Croft L., Suphioglu C., Kapat A., Athan E. (2023). Pseudomonas aeruginosa: Infections and novel approaches to treatment “Knowing the enemy” the threat of Pseudomonas aeruginosa and exploring novel approaches to treatment. Infect. Med..

[37] Tian R.-R., Zhang M.-X., Liu M., Fang X., Li D., Zhang L., Zheng P., Zheng Y.-T., Liu Y. (2020). CD24Fc protects against viral pneumonia in simian immunodeficiency virus-infected Chinese rhesus monkeys. Cell Mol. Immunol..

